# Circulating neuropeptide Y as a biomarker in postoperative atrial fibrillation cases administered off-pump coronary bypass Graft surgery

**DOI:** 10.1016/j.heliyon.2024.e31251

**Published:** 2024-05-14

**Authors:** Jian Zhang, Yuanchen He, Zongtao Yin, Rui Li, Xiaohui Zhang, Yang Wang, Huishan Wang

**Affiliations:** a Department of Cardiovascular Surgery, General Hospital of Northern Theater Command, No.83, Wenhua Road, Shenhe District, Shenyang, Liaoning,110016 China; b Postgraduate Training Base of Northern Theater Command General Hospital,Dalian Medical University, No. 9, Lvshun western south Road, LvShunKou District, Dalian, Liaoning 116044, China; c Postgraduate Training Base of Northern Theater Command General Hospital,China Medical University, No.83, Wenhua Road, Shenhe District, Shenyang, Liaoning,110016 China

**Keywords:** Neuropeptide Y, Post-Operative atrial fibrillation, Coronary artery bypass grafting, Heart rate variability

## Abstract

**Background and aims:**

Postoperative atrial fibrillation (POAF) is considered the most prevalent irregular heart rhythm after heart surgery. The cardiac autonomic nervous system significantly affects POAF, and neuropeptide Y (NPY), an abundant neuropeptide in the cardiovascular system, is involved in this autonomic regulation. The current work aimed to examine the potential association of NPY with POAF in individuals administered isolated off-pump coronary artery bypass grafting.

**Methods:**

From January 1 to May 31, 2020, we examined consecutive cases administered successful isolated off-pump coronary artery bypass grafting with no previously diagnosed atrial fibrillation (AF). Clinical characteristics and plasma samples were collected before surgery. NPY was quantified by enzyme-linked immunosorbent assay (ELISA) in peripheral blood, and POAF cases were identified through a 7-day Holter monitoring.

**Results:**

Among 120 cases with no previously diagnosed AF, 33 (27.5 %) developed POAF during hospitalization. Median NPY levels were markedly elevated in the POAF group in comparison with the sinus rhythm group (31.72 vs. 27.95, P = 0.014). Multivariable logistic regression analysis revealed age (OR = 1.135, 95%CI 1.054–1.223; P = 0.001), left atrial size (OR = 1.136, 95%CI 1.004–1.285; P = 0.043), and NPY levels in peripheral blood (OR = 1.055, 95%CI 1.002–1.111; p = 0.041) independently predicted POAF. Additionally, NPY levels were positively correlated with high-frequency (HF) (r = 0.2774, P = 0.0022) and low-frequency (LF) (r = 0.2095, P = 0.0217) components of heart rate variability.

**Conclusion:**

In summary, this study demonstrates an association between elevated NPY levels in peripheral blood before surgery and POAF occurrence.

## 1 Introduction

Postoperative atrial fibrillation (POAF) is considered the most frequent complication in patients administered heart surgery [1,2]. This condition prolongs postoperative hospital stay and increases medical expenses [3]. Furthermore, POAF has been linked to stroke, heart failure, and increased mortality [4]. Mounting evidence suggests the autonomic nervous system is involved in the induction of atrial fibrillation (AF) [5]. In a previous investigation, we observed a reduction in POAF after targeting ganglionated plexi via calcium-mediated autonomic neurotoxicity [1]. Ganglionated plexi encompass both sympathetic and parasympathetic nervous components, and the latter report did not define whether parasympathetic or sympathetic activity predominantly promotes AF. Sympathetic activity is known to enhance POAF through increased calcium transient currents [6]. Consequently, beta-blockers are widely administered before and after cardiac surgery [2,7,8]. However, beta-blockade is not highly effective in reducing POAF, suggesting the existence of alternative mechanisms.

Neuropeptide Y (NPY, 36 amino acids), produced by brain and immune (macrophages and dendritic cells) cells, can activate sympathetic nerves [[9], [10], [11], [12]]. Elevated NPY amounts were detected in myocardial infarction cases [13]. Due to the long half-life of NPY, its levels continue to rise following revascularization [13]. Notably, recent studies have identified NPY level as a potential prognostic marker of heart failure or mortality [14,15]. Animal studies indicated that NPY can attenuate cardiac remodeling by inhibiting p38/NF-κB-dependent M1 macrophage activation following acute myocardial infarction in a mouse model [10]. Additionally, NPY has been established as a biomarker of neuromodulation in AF through left atrial NPY receptors [[16], [17], [18]]. Moreover, in patients undergoing beta-blocker therapy, NPY release activates cardiac sympathetic activity [19,20]. This work aimed to assess whether circulating NPY levels could serve as a biomarker for predicting POAF in patients undergoing isolated off-pump coronary artery bypass grafting (CABG).

## 2 Materials and methods

### 2.1 Study cohort

We conducted a retrospective analysis of consecutive cases administered isolated CABG in the Department of Cardiovascular Surgery of the General Hospital of the Northern Theater Command between January 1, 2020, and May 31, 2020. Exclusion criteria were [1]: emergency surgery [2]; surgical procedure involving cardiopulmonary bypass [3]; previous heart surgery; or [4] previously diagnosed AF (as determined by 7-day Holter monitoring). Totally 120 patients were included. Each patient provided signed informed consent, and data collection and analysis were performed according to a predefined protocol that had approval from the Ethics Committee of the General Hospital of the Northern Theater Command Ethics Board. This study fully adhered to the principles outlined in the Declaration of Helsinki. Blood sample collection was carried out in the morning of the second day of hospitalization, preceding heart surgery, which was performed as described in a previous report [8].

### 2.2 POAF

As described in previous reports [1,21], AF≥30 s by any of the following methods was considered to indicate POAF: 1) routine 12-lead electrocardiography; 2) continual telemetry assessment with full disclosure from surgery completion to discharge; 3) 7-day Holter Single-lead monitoring device (Yueguang Medical Technologies, Shanghai, China) detecting cardiac arrhythmia events, applied postoperatively. Each AF event was confirmed by two physicians.

### 2.3 Heart rate variability (HRV)

Resting HRV was recorded using 7-day Holter monitoring. Time-domain indexes were determined, including the standard deviation of all normal to normal (N–N) RR intervals (SDNN), the average standard deviation of all N–N RR intervals (SDANN), the average of the standard deviations of all N–N RR intervals for each 5-min (SDNNIDX), and the root-mean square of differences between successive N–N intervals (RMSSD). Frequency-domain parameters, such as total power (TP), low frequency component (LF), high-frequency component (HF), and very low frequency (VL), were analyzed according to standard procedures.

### 2.4 Plasma NPY measurement

Plasma NPY amounts were assessed in duplicate with a specific enzyme-linked immunosorbent assay (ELISA) kit (DY8517–05, R&D, USA).

### 2.5 Statistical analysis

The statistical analysis of baseline data was performed with SPSS 24.0 (SPSS, USA). Continuous variables (age, heart rate, body-mass index, fasting blood glucose, triglyceride, cholesterol, LDL, LDH, ICU time, ventilator use time, length of hospital stay, left ventricular ejection fraction, left atrial size, LVEDV, BNP, HsTnT, HCRP, IL6, creatinine, CKMB and NPY) were presented as median (first and third quartiles) and compared by the Mann-Whitney *U* test. Categorical variates (sex, NYHA, hypertension, smoking history, left main coronary artery stenosis (>70 %), right coronary stenosis (>70 %), left anterior descending artery stenosis (>70 %), use of calcium channel blockers，use of statins and IABP) were compared by the χ2 test. Potential risk factors (P < 0.1 in univariable logistic regression analysis) were further examined by multivariable logistic regression analysis. The multivariate logistic regression method was used as a predictive model. Receiver operating characteristic (ROC) curve analysis was utilized to assess NPY and the predictive model for diagnostic sensitivity and specificity. A significance level of P < 0.05 was applied.

## 3 Results

### 3.1 Baseline clinicodemographic data

Totally 120 individuals were successfully administered isolated CABG. Postoperatively, 33 (27.5 %) patients developed POAF according to 7-day Holter results. Baseline patient features are presented in Table 1, revealing that older age (P < 0.001), increased preoperative left atrial diameter (P = 0.019), and right coronary stenosis (P = 0.035) had significant associations with elevated likelihood of POAF development.

**Table 1:** Demographic and clinical characteristics of the patients at baseline.

Characteristic	POAF（n = 33）	NO-POAF（n = 87）	P
Age（y)	66.67（64.12，69.21）	59.41（58.00，61.06）	**<0.001**
Female sex，%	11,0.33	19,0.22	0.194
NYHA = Ⅰ-Ⅲ			0.507
Ⅰ	12,0.36	27,0.31	
Ⅱ	21,0.64	57,0.66	
Ⅲ	0	3,0.03	
Heart rate	72.12(69.56，74.68)	72.63(70.89，74.38）	0.753
Hypertension，%	50,0.57	17,0.62	0.557
Smoking history，%	49,0.56	15,0.45	0.287
Body-mass index	25.16(24.06，26.25)	25.71(25.05，26.36)	0.383
Fasting blood glucose, (mmol/L)	6.19(5.65,6.73)	6.7(6.19,7.22)	0.254
Triglyceride (mmol/L)	1.76(1.46,2.07)	2.14(1.83,2.45)	0.165
Cholesterol (mmol/L)	3.97(3.65,4.28)	4.17(3.97,4.38)	0.284
LDL (mmol/L)	2.13(1.92，2.34)	2.26(2.1，2.4)	0.361
LDH (U/L)	189.45(170.84,208.07)	185.61（169.59，201.63）	0.788
ICU（h）	33.42(24.57,42.28)	35.55(27.5,43.6)	0.629
Ventilator use time（h）	23.58(19.37,27.78)	27.4(22.11,32.68)	0.300
Length of stay (day)	10.52(8.75,12.28)	11.5(10.4,12.59)	0.344
Left ventricular ejection fraction（%）	0.56(0.54,0.57)	0.55(0.54,0.57)	0.528
Left atrial size（mm）	38.79(37.19,40.38)	36.88(36.09,37.68)	**0.019**
LVEDV（ml）	103.64(95.83,111.44)	100.97(95.77,106.16)	0.570
Left main coronary artery stenosis (>70 %)	9,0.27	26,0.30	0.779
Right coronary stenosis (≥70 %)	29,0.88	60,0.69	**0.035**
Left anterior descending artery (>70 %)	33,1	86,0.99	1
Calcium channel blockers，%	18,0.55	45,0.52	0.782
Use statins，%	8,0.24	21,0.24	0.99
IABP，%	1,0.03	4,0.05	1
BNP (pg/ml)	629.01(328.97,929.05)	481.03(324.01,638.05)	0.348
HsTnT (ng/ml)	0.15(0.01,0.28)	0.06(0.03,0.09)	0.078
HCRP (mg/L)	6.6(4.13,9.07)	12.15(6.51,17.79)	0.237
IL6（pg/ml）	9.55(6.86,12.24)	8.62(6.09,11.16)	0.678
Creatinine (mg/dl)	70.64(63.92,77.36)	74(69.07,78.92)	0.458
CKMB (ng/ml)	13.21(7.08，19.33）	12.3(11.2，13.4)	0.778
NPY (pg/mL)	35.1(30.8，39.4)	29.5(28.0，31.1）	**0.002**

Values are medians (1st, 3rd quartile) or n; p < 0.05 are indicated in bold; LDL:Low Density Lipoprotein; LDH:lactate dehydrogenase; ICU:intensive care unit; LVEDV:Left ventricular end-diastolic volume; IABP:intra-aortic ballon pump; BNP:Brain natriuretic peptide; HCRP:Hypersensitive C-reactive protein; IL6:Inter Leukin 6; CKMB:CreatineKinase-MB; NPY:Neuropeptide Y.

### 3.2 NPY independently predicts POAF

**Fig. 1:**
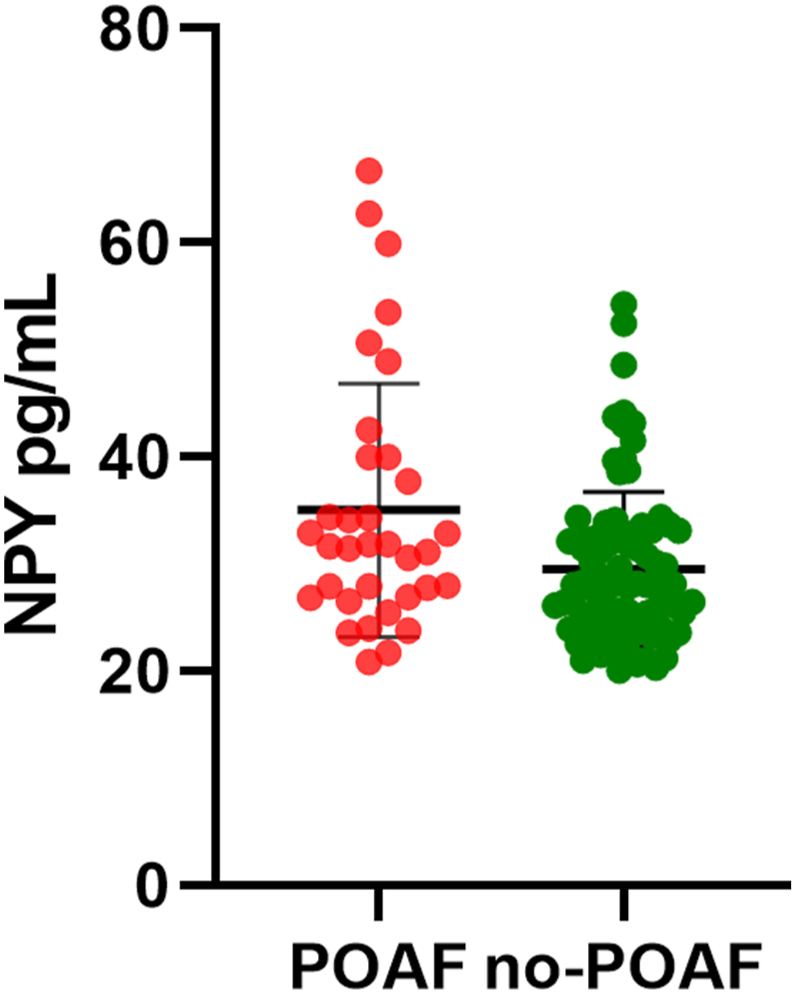
NPY levels in POAF and non-POAF patients. Medians (first and third quartiles) were compared by the Mann-Whitney *U* test (P = 0.014). Error bars represent first and third quartiles. POAF, postoperative atrial fibrillation.

Subsequently, NPY levels were assessed in peripheral blood, with significantly elevated values in POAF cases (31.72 vs. 27.95, P = 0.014) (Fig. 1). Multivariable logistic regression analysis identified age (OR = 1.135, 95%CI 1.054–1.223; P = 0.001), left atrial size (OR = 1.136, 95%CI 1.004–1.285; P = 0.043), and NPY levels in peripheral blood (OR = 1.055, 95%CI 1.002–1.111; P = 0.041) as independent risk factors for POAF (Table 2).

**Table 2:** Univariable and multivariable logistic regression of POAF.

	Uni-variable log regression			Multi-variable log regression		
	Exp(B)	95%CI	P	Exp(B)	95%CI	P
Age	1.166	1.083–1.255	<0.001	1.135	1.054–1.223	**0.001**
Left atrial size	1.126	1.016–1.248	0.024	1.136	1.004–1.285	**0.043**
Right coronary stenosis (≥70 %)	3.262	1.044–10.199	0.042	3.188	0.861–11.803	0.083
NPY	1.066	1.019–1.115	0.005	1.055	1.002–1.111	**0.041**

p < 0.05 are indicated in bold; CI:confidence interval; NPY:Neuropeptide Y.

### 3.3 Association of NPY with heart rate variability (HRV)

**Fig. 2:**
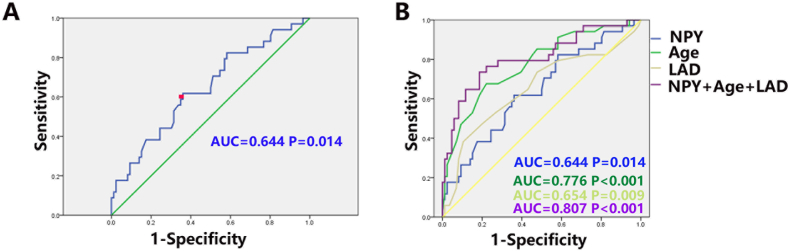
A. ROC curve analysis of the predictive value of NPY in POAF. The area under the ROC curve (AUC) was 0.64 (95 % confidence interval [CI] 0.533–0.754, P = 0.014). B. ROC curve analysis of the predictive value of combined age, left atrial size, and NPY in POAF. The AUC was 0.807 (95%CI = 0.713 to 0.901, P < 0.001).

NPY level was subjected to ROC curve analysis, yielding an area under the curve (AUC) of 0.64 (95%CI 0.533–0.754) (Fig. 2A, P = 0.014). With a cutoff point of 30.40, sensitivity and specificity for POAF prediction were 61.8 % and 64.0 %, respectively. Since NPY level had a low predictive potential, it was combined with age and left atrial size to predict POAF. As illustrated in Fig. 2B, this combination resulted in improved capability of identifying patients with POAF (AUC = 0.807, 95%CI 0.713–0.901; P < 0.001).

Patients with high NPY levels exhibited elevated lactate dehydrogenase (LDH), left atrial size, and amounts of inflammation markers (CRP and IL-6) (Table 3).

**Table 3:** Patient characteristics by high versus low NPY levels.

Characteristic	NPY Low（n = 68）	NPY High（n = 52）	P
Age（y)	60.28(58.43,62.13)	63.10(60.87,65.32)	0.052
Female sex，%	15，0.22	15，0.29	0.395
NYHA = Ⅰ-Ⅲ			
Ⅰ	24，0.35	15，0.29	0.201
Ⅱ	41，0.60	37，0.71	
Ⅲ	3，0.04	0，0	
Heart rate	71.81(69.77,73.85)	73.22(71.21,75.22)	0.281
Hypertension，%	37，0.54	30，0.58	0.720
Smoking history，%	36，0.53	28，0.54	0.922
Body-mass index	25.63(24.85,26.40)	25.38(24.56,26.19)	0.784
Fasting blood glucose, (mmol/L)	6.56(6.04,7.09)	6.58(5.95,7.22)	0.995
Triglyceride (mmol/L)	2.14(1.79,2.48)	1.92(1.58,2.26)	0.358
Cholesterol (mmol/L)	4.14(3.92,4.36)	4.10(3.82,4.38)	0.765
LDL (mmol/L)	2.23(2.07,2.40)	2.21(2.00,2.41)	0.863
LDH (U/L)	170.76(161.30,180.23)	209.12(183.18,235.06)	**0.009**
ICU（h）	31.75(24.89,38.61)	39.24(27.68,50.79)	0.163
Ventilator use time（h）	26.24(20.59,31.88)	26.47(20.85,32.09)	0.574
Length of stay (day)	11.84(10.39,13.29)	10.41(9.48,11.34)	0.128
Left ventricular ejection fraction（%）	0.554(0.539,0.569)	0.555(0.540,0.570)	0.980
Left atrial size（mm）	36.51(35.72,37.31)	38.56(37.29,39.83)	**0.005**
LVEDV（ml）	98.65(93.27,104.03)	105.58(98.70,112.45)	0.109
Left main coronary artery stenosis (>70 %)	20，0.29	15，0.29	0.946
Right coronary stenosis (≥70 %)	52，0.76	37，0.71	0.510
Left anterior descending artery (>70 %)	68，1	51，0.98	0.893
Calcium channel blockers，%	37，0.54	26，0.50	0.632
Use statins，%	16，0.24	13，0.25	0.852
IABP，%	2，0.03	3，0.06	0.759
BNP (pg/ml)	426.55(266.21,586.89)	573.13(373.69,772.56)	0.121
HsTnT (ng/ml)	0.048(0.012,0.083)	0.14(0.047,0.224)	0.071
HCRP (mg/L)	6.04(3.68,8.40)	16.62(7.66,25.57)	**0.026**
	6.74(5.20,8.27)	11.68(7.66,15.69)	**0.024**
Creatinine (mg/dl)	72.00(67.79,76.22)	73.89(66.32,81.46)	0.568
CKMB (ng/ml)	11.54(10.55,12.53)	14.00(9.91,18.08)	0.264
POAF	13，0.19	20，0.38	**0.019**

Values are medians (1st, 3rd quartile) or n; p < 0.05 are indicated in bold; NPY:Neuropeptide Y; LDL:Low Density Lipoprotein; LDH:lactate dehydrogenase; ICU:intensive care unit; LVEDV:Left ventricular end-diastolic volume; IABP:intra-aortic ballon pump; BNP:Brain natriuretic peptide; HCRP:Hypersensitive C-reactive protein; IL6:Inter Leukin 6; CKMB:CreatineKinase-MB; .

**Fig. 3:**
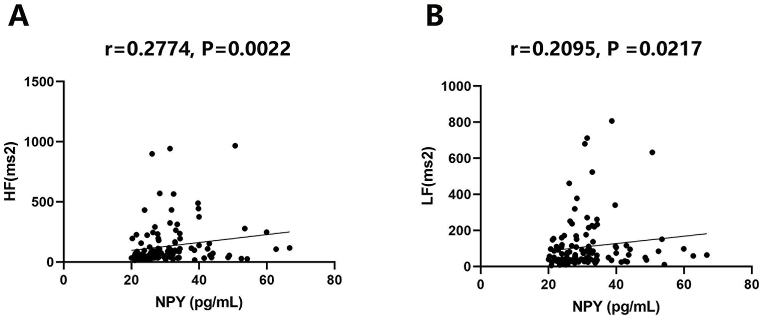
NPY is associated with HF and LF. A. Correlation between NPY and HF (r = 0.2774, P = 0.0022); B. Correlation between NPY and LF (r = 0.2095, P = 0.0217).

Furthermore, the associations of NPY levels with postoperative HRV data obtained from 7-day Holter monitoring were examined. Table 4 reveals that LF (P = 0.016) and HF (P = 0.005) were associated with NPY. Other HRV indexes, including SDNN, SDANN, SDNNIDX, RMSSD, percentage of the interval differences of successive R–R intervals greater than 50 ms (PNN50), total power (TP), very low frequency (VLF) and LF/HF, were not significantly associated with POAF (Table 4). NPY exhibited significant correlations with HF (r = 0.2774, P = 0.0022) and LF (r = 0.2095, P = 0.0217) (Fig. 3 A and B). Interestingly, HF was also starkly increased in POAF patients in comparison with non-POAF cases (P < 0.001), while LF/HF was markedly decreased (Supplementary Table 1, P = 0.047).

**Table 4:** NPY and HRV.

Characteristic	NPY low（n = 68）	NPY high（n = 52）	P
SDNN（ms）	51.47(48.44,54.49)	54.95(51.32,58.58)	0.110
SDANN（ms）	51.47(48.44,54.49)	54.95(51.32,58.58)	0.089
SDNNIDX	19.43(17.91,20.95)	20.77(18.85,22.68)	0.249
RMSSD（ms）	17.46(15.95,18.98)	19.80(17.31,22.30)	0.106
PNN50（%）	2.95(2.19,3.72)	3.78(2.40,5.17)	0.286
TP (ms2)	3852.41(3393.16,4311.67)	4700.49(3900.29,5500.69)	0.057
LF (ms2)	78.82(58.11,99.54)	150.39(96.64,204.14)	**0.016**
HF (ms2)	101.78(68.56,135.00)	174.96(116.80,233.11)	**0.005**
VLF (ms2)	273.49(214.69,332.29)	382.77(254.21,511.34)	0.107
LF/HF	115.30(93.93,136.67)	104.80(83.22,126.38)	0.500

Values are medians (1st, 3rd quartile); SDNN:the standard deviation of all normal-to-normal RR interval; SDANN:the average standard deviation of all normal-to-normal RR interval; SDNNIDX:the mean value of the standard deviation of all normal-to-normal RR intervals for all 5-min segments of the entire registration; RMSSD:The root-mean square of differences between successive normal to normal intervals; PNN50:Percentage of the interval differences of successive R–R intervals greater than 50 ms; TP:Total Power; LF:Low frequency component; HF:High-frequency component; VLF: very low frequency.

## 4 Discussion

In this study, NPY levels in peripheral blood were associated with POAF in cases administered off-pump CABG. Additionally, NPY levels were correlated with increased LDH, left atrial size, amounts of inflammatory markers, and HRV parameters such as LF and HF.

It is well established that various cells, including lymphocytes, macrophages, and nervous system cells, release NPY [10]. NPY binds to Y-receptors, activating the NPY signaling pathway involved in multiple pathological processes [9]. Evidence indicates that heightened NPY levels are associated with impaired microvascular function, enhanced myocardial injury, and decreased ejection fraction following myocardial infarction, mediated by the Y1 receptor [22]. Similar findings show that NPY levels are correlated with microvascular function and infarct size following ST-segment–elevation myocardial infarction (STEMI) [14]. A recent study revealed elevated cardiac NPY amounts in the acute phase of subarachnoid hemorrhage, with NPY acting on Y1 receptors to predispose to ventricular arrhythmias [23]. Interestingly, NPY triggers ventricular arrhythmias via the Y1 receptor in STEMI even after beta-blocker use [20]. Given the widespread use of beta-blockers upon heart surgery, POAF prevalence remains high, at nearly 30 % [1]. In AF patients, NPY levels and NPY receptors are associated with AF progression [17]. In 2007, an increase in NPY was detected in 83 patients administered on-pump CABG, although not statistically significant [24]. In the current study involving 120 patients undergoing off-pump CABG, elevated NPY levels in peripheral blood before surgery were correlated with POAF, suggesting the involvement of neuromodulation in AF initiation.

As shown above, NPY was associated with LDH, left atrial size, and inflammation. Studies investigating the causal relationship between NPY and LDH or left atrial size are scarce. However, the relationship between NPY and inflammation has been widely discussed. Lan and colleagues reported NPY deficiency inhibits inflammation in mice with experimental acute kidney injury and acute myocardial infarction by inactivating M1 macrophages [10,12]. In the central nervous system, NPY inhibits IL-1β and TNF-α production in microglial cells [25]. Additionally, evidence indicates a role for NPY in aging for its anti-inflammatory properties [26]. On the contrary, in patients administered CABG, NPY was positively corelated with IL-6 and CRP, indicating inflammation is not the only mechanism.

Mounting evidence supports a crucial role for the autonomic nervous system in AF induction [27,28]. Both parasympathetic and sympathetic stimuli might independently induce AF, but joint parasympathetic and sympathetic discharges are particularly efficient inducers of AF, because of the combined effects of reduced atrial effective refractory period and elevated calcium transient currents [5]. Interestingly, changes in HRV prior to AF initiation are consistent with joint sympathetic discharges [5,29,30]. On the other hand, the autonomic nervous system was applied to prevent POAF by suppressing autonomic system overdrive and downregulating inflammation [31]. Therefore, many therapies targeting the autonomic nervous system for AF and POAF prevention are available. Our previous clinical study further demonstrated that calcium-induced autonomic denervation in ganglionated plexi prevents POAF [1]. Consistent with these findings, targeting the sympathetic nervous system with moxonidine, a drug with centrally sympathoinhibitory activity, has shown promise in reducing post-ablation AF recurrence [32]. Additionally, stimulating the vagus nerve has demonstrated potential in preventing POAF in randomized clinical trials [33], although its efficacy needs confirmation in future studies. A more extensive investigation into stimulating the vagus nerve for POAF prevention is required to establish its potential effectiveness.

HRV reflects the dynamics of the autonomic nervous system, modulated by autonomic control of the heart. Different HRV levels are related to sympathetic and parasympathetic dominance during high attention, arousal, and stress [34]. The main frequency domains in HRV are LF and HF. Parasympathetic activity represents the main contributing factor of the HF component, while LF reflects both sympathetic and parasympathetic activities [35]. Our findings indicate a significant correlation between high NPY levels and elevated HF and LF, highlighting that parasympathetic activity is involved in NPY production. Consistent with our findings, NPY polymorphism was shown to modulate vagal outflow to the heart under high chronic stress [36]. Mechanistically, NPY-Y2 receptors found on vagal neurons regulate sympatho-vagal cross-talk [37]. Vagal AF, often occurring without structural heart disease, tends to be maintained by shortening the atrial effective refractory period [38,39]. Furthermore, the present study found that increased HF was associated with POAF, implying high NPY levels may stimulate vagal neurons, which contributes to POAF maintenance.

## Limitations

The major limitations of this research include its retrospective design and the small population enrolled in a single center. Another limitation was the relatively older age in the examined population, as vagal HRV decreases with age [40]. This limitation may constrain the potential correlation between NPY and HRV.

In conclusion, this study demonstrates a significant increase in NPY levels before surgery in patients who develop AF post-CABG. High NPY levels independently predict POAF, and the above data suggest an association between NPY levels and HRV.

## Ethics statement

The Ethics Committee of the General Hospital of Northern Theater Command Ethics Board approved this study (No. 2019-38).

## Funding

The current study was funded by the National Natural Sciences Fund Project of China (81970310, 82070239 and 82170328), the Provincial Key R&D Program (2021JH2/10300082 and 2022JH2101500030) and the Liaoning Revitalization Talents Program (XLYC2203117).

## Data availability statement

The essential data are available from the corresponding author upon reasonable request.

## CRediT authorship contribution statement

**Jian Zhang:** Writing – original draft, Investigation, Conceptualization. **Yuanchen He:** Resources, Methodology, Investigation, Data curation. **Zongtao Yin:** Validation, Resources, Methodology, Investigation. **Rui Li:** Validation, Investigation. **Xiaohui Zhang:** Investigation, Data curation. **Yang Wang:** Resources. **Huishan Wang:** Writing – review & editing, Supervision, Conceptualization.

## Declaration of competing interest

The authors declare that they have no known competing financial interests or personal relationships that could have appeared to influence the work reported in this paper.
