# Supplementary material for: Circulating neuropeptide Y as a biomarker in postoperative atrial fibrillation cases administered off-pump coronary bypass Graft surgery
Source: Heliyon. 2024 May 14;10(10):e31251. doi: 10.1016/j.heliyon.2024.e31251 (PMC11129009; doi:10.1016/j.heliyon.2024.e31251)
Supplement: Multimedia component 1 [file mmc1.docx]

Table Supplementary 1.HRV and POAF.

| Characteristic |  | POAF（n=33） | NO-POAF（n=87） | P |
| --- | --- | --- | --- | --- |
| SDNN（ms） |  | 57.60(49.25,63.70) | 53.80(46.90,68.00) | 0.679 |
| SDANN（ms） |  | 55.50(45.45,61.35) | 48.60(42.90,61.80) | 0.331 |
| SDNNIDX |  | 18.50(15.10,24.25) | 18.90(15.30,24.20) | 0.742 |
| RMSSD（ms） |  | 17.60(14.30,26.30) | 15.20(12.60,20.90) | 0.085 |
| PNN50（%） |  | 2.30(1.05,6.05) | 1.50(0.70,4.30) | 0.118 |
| TP (ms2) |  | 4210.30(2875.25,5508.65) | 3390.90(2399.50,5019.30) | 0.283 |
| LF (ms2) |  | 64.50(35.90,163.75) | 50.50(29.80,108.83) | 0.217 |
| HF (ms2) |  | 111.50(74.95,215.30) | 55.70(34.40,99.40) | **＜0.001** |
| VLF (ms2) |  | 205.80(102.35,434.65) | 196.70(112.00,408.90) | 0.979 |
| LF/HF |  | 61.50(47.45,126.65) | 90.10(62.70,148.80) | **0.047** |

Values are medians (1st, 3rd quartile); SDNN:the standard deviation of all normal-to-normal RR interval; SDANN:the average standard deviation of all normal-to-normal RR interval; SDNNIDX:the mean value of the standard deviation of all normal-to-normal RR intervals for all 5-min segments of the entire registration; RMSSD:The root-mean square of differences between successive normal to normal intervals; PNN50:Percentage of the interval differences of successive R-R intervals greater than 50ms; TP:Total Power; LF:Low frequency component; HF:High-frequency component; VLF: very low frequency.
